# 非小细胞肺癌13组、14组淋巴结转移规律探讨

**DOI:** 10.3779/j.issn.1009-3419.2017.04.04

**Published:** 2017-04-20

**Authors:** 磊 张, 吉雅 布仁, 宇飞 王, 巴特尔 韩, 占林 郭

**Affiliations:** 010050 呼和浩特，内蒙古医科大学附属医院胸外科 Department of Thoracic Surgery, The Affiliated Hospital of Inner Mongolia Medical University, Huhhot 010050, China

**Keywords:** 肺肿瘤, 淋巴结, 转移, Lung neoplasms, Lymph node, Metastasis

## Abstract

**背景与目的:**

淋巴结转移是影响肺癌肿瘤-淋巴结-转移（tumor-node-matastasis, TNM）分期的重要因素之一，在非小细胞肺癌（non-small cell lung cancer, NSCLC）患者的手术中，13组、14组淋巴结因其隐藏于肺叶的深部而忽视做病理检测，影响术后病理分期准确性。本研究旨在探讨13组、14组淋巴结在NSCLC术中的阳性检出率及其对病理分期的影响。

**方法:**

选取内蒙古医科大学附属医院100例NSCLC手术患者为研究对象，剖取胸内2组-12组、第13、14组淋巴结行病理检测，分析肿瘤的大小、部位、病理类型等因素与胸内淋巴结转移率的关系。

**结果:**

100例患者胸内淋巴结转移率为47.0%，10组-12组、N2淋巴结、13组、14组淋巴结阳性率有统计学差异（*P* < 0.05）；不同T分期13组、14组淋巴结漏检率有统计学差异（*P* < 0.05）；周围型与中央型NSCLC的N1期漏检率无统计学差异（*P*>0.05）；不同病理类型肿瘤之间N1期漏诊率无统计学差异（*P*>0.05）。此外，发现有12例患者存在非肿瘤所在叶、段支气管旁淋巴结转移。

**结论:**

临床上检测NSCLC 13组、14组与非肿瘤所在叶支气管旁淋巴结的转移情况十分必要，有利于获取术后准确的TNM分期，对于指导术后治疗意义重大。

## Abstract (en)

**Background and objective:**

Lymph node metastasis is one of the important factors affecting the tumor-node-matastasis (TNM) staging of lung cancer. In patients with surgery, 13 groups and 14 groups of lymph nodes ignored because of the deep hidden in the lung. In this paper, the positive detection rate of 13 groups and the 14 groups of lymph nodes in non-small cell lung cancer (NSCLC) and their effects on pathological stage were studied.

**Methods:**

100 cases of NSCLC were collected from the Affiliated Hospital of Inner Mongolia Medical University as the research object, cut out the intrathoracic 2-12 group, 13, 14 lymph node metastasis rate for pathological examination, relationship factors of statistical analysis of the size of primary tumor, location, pathological type and lymph node.

**Results:**

100 cases of patients with intrathoracic lymph node metastasis rate was 47.0%, with the 10-12 groups lymph node, N2 lymph nodes, 13, 14 groups lymph node positive rate had significant differences (*P* < 0.05); T stage 13, 14 groups of lymph node detection rate has statistically significant difference (*P* < 0.05); peripheral and central NSCLC stage N1 undetected rate no statistical difference (*P*>0.05); various pathological types of tumors between N_1_ missed rate had no significant difference (*P*>0.05). In addition, 12 patients were found to have non-tumor-derived lobe lymph node metastasis.

**Conclusion:**

It is necessary to detect the metastatic lymph nodes of 13, 14 group and non-tumor-derived lobe in NSCLC. It is helpful to obtain accurate TNM staging and to guide postoperative treatment.

肺癌是我国发病率和死亡率最高的恶性肿瘤^[1]^，非小细胞肺癌（non-small cell lung cancer, NSCLC）是肺癌的主要类型，治疗方法是以手术为主的综合治疗，其术后5年生存率仅为24.4%^[2]^。淋巴结转移是NSCLC最常见的转移途径，也是影响预后和分期的一个最重要因素。全面、准确检测淋巴结的病理状态可以精确地进行术后肿瘤-淋巴结-转移（tumor-node-matastasis, TNM）分期、指导治疗、判断预后，对提高肺癌生存率和改善患者生活质量具有十分重要的临床意义。目前，国内外对NSCLC的N2淋巴结的研究较多，对N1淋巴结转移，尤其是肺内13组、14组淋巴结和非原发肿瘤所在叶、段淋巴结转移，相关研究文献较少。因此，本研究探讨NSCLC肺叶内13组、14组淋巴结及非肿瘤所在叶、段支气管旁淋巴结的转移情况，以提高术后TNM分期的准确性。

## 1 资料与方法

### 1.1 研究对象

2012年4月-2013年12月内蒙古医科大学附属医院胸外科共完成NSCLC根治性手术治疗的患者112例。其中，100例患者符合入组标准，男性70例，女性30例，年龄31岁-75岁，平均年龄58.7岁；按国际抗癌联盟（Union for International Cancer Control, UICC）第七版TNM分期，术后的TNM分期：Ⅰa期12例，Ⅰb期15例，Ⅱa期22例，Ⅱb期17例，Ⅲa期33例，Ⅲb期1例。患者的一般资料见表 1。

**1:** 患者一般临床资料（*n*=100） The general clinical data of patients (*n*=100)

	Cases	Percentage (%)
Primary site		
Upper lobe of right lung	33	33.0
Middle lobe of right lung	5	5.0
Lower lobe of right lung	29	29.0
Upper lobe of left lung	18	18.0
Lower lobe of left lung	15	15.0
Pathologic type		
Squamous carcinoma	54	54.0
Adenocarcinoma	37	37.0
Other types	9	9.0
Clinical classification		
Central type	43	43.0
Peripheral type	57	57.0
T staging		
T1	22	22.0
T2	50	50.0
T3	26	26.0
T4	2	2.0
N staging		
N0	47	47.0
Group 10-12	15	15.0
N2	25	25.0
Group 13, 14	13	13.0

### 1.2 研究方法

#### 1.2.1 入组标准

① 术后病理明确诊断为NSCLC者；②术前未接受过放疗或化疗者；③术前均行胸部计算机断层扫描（computed tomography, CT）、头部CT或磁共振成像（magnetic resonance imaging, MRI）、腹部B超或CT、发射型计算机断层扫描（emission computed tomography, ECT）等影像学检查，检查结果提示患者无远处转移，有手术指征，且患者心肺功能可耐受手术治疗。④手术行肺叶或全肺切除，系统淋巴结清扫者。

#### 1.2.2 手术方法

所有患者均行开胸手术治疗，根据肿瘤部位与侵犯范围，采取不同的手术方式，术式包括肺叶切除、全肺切除、部分伴血管或/和支气管成形，均行淋巴结系统清扫。

#### 1.2.3 淋巴结的采集方法

依据Mountain/Naruke淋巴结图谱，N2淋巴结包括2组-9组淋巴结；N1淋巴结包括10组-14组淋巴结^[3]^，第13组淋巴结是指段支气管周围淋巴结，第14组淋巴结是指亚段支气管周围淋巴结。

2组-12组淋巴结由术者在术中常规清扫，术中同时注重清扫非肿瘤所在肺叶、段的淋巴结，肺内非原发肿瘤所在叶、段支气管旁淋巴结分别单独记录送检，以便与原发肿瘤所在叶、段支气管旁淋巴结区分。

13组、14组淋巴结在肺叶切除离体后剖取，具体方法：将切除的肺叶放置在标本盘中，确定肿瘤所在位置，剪断结扎的肺动、静脉断端，肝素生理盐水浸泡标本约5 min后，轻轻挤压使肺积血排净，清洗标本表面血液，辨认叶支气管断端，沿支气管走行方向解剖，使支气管树完全显露，其间清扫所有肉眼可见淋巴结。

### 1.3 统计学处理

数据分析采用SPSS 19.0统计软件处理，频率分析采用*χ*^2^检验，以*P* < 0.05为有统计学差异。

## 2 结果

### 2.1 全组清扫淋巴结个数与转移率

全组共清扫淋巴结2, 723枚，平均每例患者27.2枚。清扫10组-12组淋巴结699枚，转移82枚，转移率11.7%；13组淋巴结416枚，转移49枚，转移率为11.8%；14组淋巴结199枚，转移12枚，转移率为6.0%；清扫N2淋巴结1, 409枚，转移88枚，转移率为6.3%。10组-12组、N2、13组、14组淋巴结阳性率差异有统计学意义（*χ*^2^=25.947, *P* < 0.001），10组-12组、13组的淋巴结转移率明显高于N2、14组，见表 2。

**2:** 清扫淋巴结个数、转移个数与转移率 The number of lymph nodes, transfer number and the transfer rate

	Number	Transfer number	Transfer rate	
Group 10-12	699	82	11.7%	*χ*^2^=25.947，*P* < 0.001
N2	1, 409	88	6.3%	
Group 13	416	49	11.8%	
Group 14	199	12	6.0%	

### 2.2 单纯13组、14组淋巴结转移情况与漏诊率

#### 2.2.1 单纯13组、14组淋巴结转移情况

全组共有13组、14组淋巴结转移者30例，其中，有10组-12组和/或N2淋巴结转移，同时伴有13组、14组淋巴结转移者17例；无10组-12组、N2淋巴结转移，仅有13组、14组淋巴结转移（即单纯13组、14组转移）者13例。单纯13组、14组转移率与不同的临床分型、T分期、肿瘤原发部位、病理类型无相关性（表 3、表 4）。

**3:** 单纯13组、14组淋巴结转移病理特点 Pathologic characteristics of lymph node metastasis in groups 13, 14

	Cases	Cases of lymph node metastasis in groups 13, 14	Transfer rate	*χ*^2^	*P*
Clinical classification				2.095	0.148
Central type	43	8	18.6%		
Peripheral type	57	5	8.8%		
T staging				7.226	0.065
T1	22	4	18.2%		
T2	50	8	16.0%		
T3	26	0	0		
T4	2	1	50.0%		
Primary site				2.564	0.633
Upper lobe of right lung	33	3	9.1%		
Middle lobe of right lung	5	0	0		
Lower lobe of right lung	29	4	13.8%		
Upper lobe of left lung	18	4	22.2%		
Lower lobe of left lung	15	2	13.3%		

**4:** 不同病理类型的单纯13组、14组淋巴结转移率 The transfer rate of lymph node metastasis in groups 13, 14 of different pathological types

	Cases	Cases of lymph node metastasis in groups 13, 14	Transfer rate	*P*
Central type				0.445
Squamous carcinoma	30	7	23.3%	
Adenocarcinoma	10	1	10.0%	
Other	3	0	0	
Peripheral type				0.701
Squamous carcinoma	23	2	8.7%	
Adenocarcinoma	28	3	10.7%	
Other	6	0	0	

#### 2.2.2 13组、14组淋巴结转移的漏诊率

本组中，若不剖取检测13组、14组淋巴结，则N0患者有28例，而13组、14组淋巴结病理检测结果显示：发生单纯13、14组淋巴结转移患者13例；N1期漏诊率为46.4%（13/28）。统计分析N1期漏诊率在不同临床病理特征的差异，结果发现：不同T分期中，T1、T2、T3、T4的N1期漏诊率具有统计学差异（*χ*^2^=8.640, *P*=0.034）；而不同的病理类型、肿瘤部位，N1期漏诊率无统计学差异（表 5）。在全组患者中，因单纯13组、14组淋巴结转移致患者术后TNM分期提高者8例，分别为：Ⅰa提高到Ⅱa期4例，Ⅰb期提高到Ⅱa期3例，Ⅱa期提高到Ⅱb期1例。

**5:** 不同临床病理特征的N1期漏诊率 The N1 phase missed diagnosis rate of different clinicopathological features

Factor	Cases	N0	10-12	N2	13/14	N1 phase missed diagnosis rate	*P*
Pathologic type							0.615
Squamous carcinoma	53	24	9	11	9	50.0% (9/18)	
Adenocarcinoma	38	16	5	13	4	44.4% (4/9)	
Other	9	7	1	1	0	0 (0/1)	
Clinical classification							0.778
Central type	43	14	10	11	8	44.4% (8/18)	
Peripheral type	57	33	5	14	5	50.0% (5/10)	
T staging							0.034
T1	22	12	1	5	4	80.0% (4/5)	
T2	50	24	7	11	8	53.3% (8/15)	
T3	26	11	6	9	0	0 (0/6)	
T4	2	0	1	0	1	50.0% (1/2)	
Primary site							0.202
Upper lobe of right lung	33	14	6	10	3	33.3% (3/9)	
Middle lobe of right lung	5	4	0	1	0	0 (0/0)	
Lower lobe of right lung	29	12	3	10	4	57.1% (4/7)	
Upper lobe of left lung	18	7	5	2	4	44.4% (4/9)	
Lower lobe of left lung	15	10	1	2	2	66.7% (2/3)	

### 2.3 非肿瘤所在叶、段支气管旁淋巴结转移情况

本组100例患者中，手术同时清扫了76例相邻肺叶11组、12组淋巴结，发现12例患者有非肿瘤所在叶、段支气管旁淋巴结转移（表 6），其中，有3例患者仅有非肿瘤所在叶、段支气管旁淋巴结转移，其他的肺内、纵隔淋巴结均未发现淋巴结转移。3例患者的TNM分期均被上调，调整后的TNM分期分别为T2aN1M0、T3N1M0、T2aN1M0，包括中央型2例，周围型1例；腺癌2例，鳞癌1例。

**6:** 非肿瘤所在叶支气管旁淋巴结转移情况 The transfer situation of non-tumor adjacent lymph nodes

Clinical pathological characteristics	With NTBL	Without NTBL
Pathologic type		
Squamous carcinoma	3	48
Adenocarcinoma	9	16
Other clinical classification	0	0
Central type	8	37
Peripheral type	4	27
N staging		
N1	4	47
N2	8	17
NTBL is the non-neoplastic bronchial lymph node metastasis.		

## 3 讨论

目前，NSCLC的手术治疗对胸内淋巴结清扫较为完全，但较少关注肺内的淋巴结，尤其是肺叶深部的第13组、14组淋巴结及非肿瘤所在肺叶支气管旁淋巴结转移情况^[4]^，导致部分N1患者漏诊，降低了部分患者的TNM分期。由于Ⅰa期的NSCLC患者不被推荐术后化疗或放疗^[5]^，这样就使此类患者术后无法得到正确的治疗。

吴楠等^[6]^剖取了90例NSCLC患者切除肺叶标本内的13组、14组淋巴结，并进行病理学检查分析，结果发现N0期诊断准确率仅为77.8%，N1期的漏诊率高达44.4%，这与本研究的N1期漏诊率为46.4%基本一致。本研究发现不同T分期NSCLC，N1期漏诊率也有不同，T1、T2期漏诊率明显高于T3、T4期，提示N1期淋巴结在肿瘤较小的时候可能就已经发生肺内淋巴结转移。此外，本研究结果显示N1期的漏诊率与组织学分型、临床分型、原发肿瘤所在叶无关，这与贺政等^[7]^的研究结果类似。

曹磊等^[8]^对52例肺癌根治性手术患者术后淋巴结病理进行研究，发现不同组的淋巴结转移率有显著差异。Naruke等^[9]^的一项1, 815例NSCLC患者术后病理检测结果显示，第13组淋巴结转移率为8.4%-17.6%，第14组淋巴结转移率为2.5%-4.7%。本研究发现，10组-12组、N2、13组、14组淋巴结的转移率分别为11.7%、6.3%、11.8%、6.0%，且10组-12组、13组淋巴结转移率显著高于N2、14组淋巴结的转移率，可能的原因是14组淋巴结在肺内的位置深在，有时与13组淋巴结相互融合，14组淋巴结的取材较13组困难；也可能与本组中央型、周围型患者所占比例基本相当有关。可以看出，不论中央型还是周围型患者，13组、14组淋巴结均可能出现转移，提示我们在临床上应该注重13组、14组淋巴结的剖取和病理检测。

本研究中，单纯第13组、14组淋巴结转移率在不同的病理分类无明显差别，Yamanaka等^[10]^报告周围型T1期肿瘤单纯13组、14淋巴结转移率为10.6%，本组病例T1期单纯13组、14组淋巴结转移率为18.2%，周围型T1期肿瘤单纯13组、14组淋巴结转移率为9.1%（2/22），与国外报道资料基本一致。

宋平平等^[11]^的研究发现，非肿瘤所在肺叶的淋巴结转移率为19.44%；国外Scott等^[12]^的研究发现非原发肿瘤所在叶支气管旁淋巴结转移率为19.0%，本组非肿瘤所在肺叶的淋巴结转移率为15.8%，均与上述结果基本一致。此外，本组有3例患者只有非肿瘤所在叶支气管旁淋巴结转移，其他的肺内、纵隔淋巴结（包括13组、14组淋巴结）均未发现淋巴结转移，提示在肺癌手术中，一定要重视系统的淋巴结清扫，同时也要注重相邻肺叶淋巴结的清扫。
